# Indoor Air Quality including Respiratory Viruses

**DOI:** 10.3390/toxics9110274

**Published:** 2021-10-21

**Authors:** Antonio López, Esther Fuentes, Vicent Yusà, F. Xavier López-Labrador, Marisa Camaró, Cristina Peris-Martinez, Martin Llácer, Susana Ortolá, Clara Coscollà

**Affiliations:** 1 FISABIO-Public Health, Foundation for the Promotion of Health and Biomedical Research in the Valencian Region, 21, Avenida Catalunya, 46020 Valencia, Spain; lopez_anttob@gva.es (A.L.); fuentes_estfer@gva.es (E.F.); yusa_vic@gva.es (V.Y.); F.Xavier.Lopez@uv.es (F.X.L.-L.); 2 Public Health Laboratory of Valencia, 21, Avenida Catalunya, 46020 Valencia, Spain; camaro_mar@gva.es (M.C.); llacer_marlun@gva.es (M.L.); ortola_sus@gva.es (S.O.); 3 Analytical Chemistry Department, University of Valencia, Edifici Jeroni Muñoz, Dr. Moliner 50, 46100 Burjassot, Spain; 4 Microbiology Department, Faculty of Medicine, University of Valencia, Av. de Blasco Ibáñez, 46010 Valencia, Spain; 5 CIBERESP, Instituto de Salud Carlos III (Institute of Health Carlos III), Av. Monforte de Lemos, 5, 28029 Madrid, Spain; 6 Foundation for the Promotion of Health and Biomedical Research in the Valencia Region, FISABIO-Mediterranean Ophthalmological Foundation (FOM), 12, Avenida Pío Baroja, 46015 Valencia, Spain; peris_crimar@gva.es; 7 Surgery Department (Ophthalmology), Faculty of Medicine, University of Valencia, 17, Avenida Blasco Ibáñez, 46010 Valencia, Spain

**Keywords:** SARS-CoV-2, air, sampling methodology, respiratory virus, indoor air quality

## Abstract

The detection of SARS-CoV-2 in indoor environments is a cause of increasing concern. In this study, three sampling methodologies have been used in order to collect SARS-CoV-2 and 17 other respiratory viruses in indoor air, combined with a new analytical process to analyze respiratory viruses. Different areas of an ophthalmological hospital were investigated for the presence of these airborne viruses. Moreover, indoor air quality (IAQ) parameters (carbon dioxide, CO_2_; carbon monoxide, CO; nitrogen dioxide, NO_2_; volatile organic compounds, VOCs; formaldehyde, HCHO; and particulate matter, PM) have been examined to study the relationship between IAQ and airborne viruses. All indoor air and surface samples assessed were found to be negative for SARS-CoV-2. Nevertheless, another airborne respiratory virus (HRV/ENV) was detected, illustrating that the methodology set out here is a suitable one. Regarding the results for the IAQ, chemical parameters studied in the hall and waiting room of the hospital presented acceptable values. However, in the doctor′s consultation room VOCs and HCHO show some instantaneous levels higher than the recommended guide values. The methodological approach described in this paper, integrating conventional IAQ and the assessment of bioaerosols, can be used in research and control programs aimed at promoting a healthy indoor environment.

## 1. Introduction

COVID-19 disease has rapidly spread throughout the world since it was first detected in December 2019 in Wuhan (China). Concerned by the alarming levels of spread and severity of the disease worldwide, the World Health Organization (WHO) stated in March 2020 that COVID-19 could be characterized as a pandemic [1]. In Spain, around 70,000 people died and more than 3 million were infected by February 2021, which corresponds to the sampling period of the present study [2]. SARS-CoV-2 can be transmitted through the following modes: (i) particles emitted from breathing; (ii) droplets greater than 100 µ; (iii) aerosols (particles smaller than 100 µm); and (iv) fomites (contact with surfaces contaminated with the virus that we touch and they can spread to our eyes, nose, or mouth). Nevertheless, there is consolidated evidence that one of the main modes of SARS-CoV-2 transmission is through inhalation of aerosols [3,4,5,6]. In fact, in indoor environments, SARS-CoV-2 can be transmitted through aerosols at a distance of more than two meters between people, given that they remain in the air and accumulate if the area is not properly ventilated [6]. Moreover, Van Doremalen et al. (2020) [4] pointed out that the coronavirus can be airborne for 3 h, while other recent studies have detected concentrations of SARS-CoV-2 in the indoor air of hospitals [7,8,9,10]. 

Apart from SARS-CoV-2, there are other airborne respiratory viruses which can spread via similar main transmission routes to SARS-CoV-2, namely direct contact, respiratory droplets, and airborne transmission. However, whereas infection control measures (e.g., hand washing and wearing face masks) can reduce the first two modes, the third route, airborne transmission, is difficult to prevent since respiratory viruses are ubiquitous in the environment, with virus particles constantly circulating in the air [11]. Airborne transmission of other respiratory viruses, such as influenza or rhinovirus, has already been studied in hospitals [12,13] since aerosol transmission has been demonstrated as being the most probable mode of transmission for adults.

Nowadays, a standard procedure does not exist for collecting SARS-CoV-2 and other respiratory viruses in the ambient air. Recently, Rahmani et al., 2020 [14] and Pena et al., 2021 [15] published two reviews about the most widely used collection methods in the literature, highlighting four sampling methodologies: (i) a Polytetrafluoroethylene (PTFE, 0.3 µm) filter inserted in 3-stage cassettes; (ii) Biosampler that bubbles the aerosol in a liquid (using impinge); (iii) an MD-8 air scan sampler, using gelatin filters; and (iv) Cyclone samplers. These sampling methodologies differ not only in their technology, but also in their flow rates, collection time, and total volume of air collected. In general, the sampling time varies from a few minutes (15–20 min) to 4 h, at flow rates of between 1 L/min and 150 L/min, with the air volume ranging from 0.06 m^3^ to 1.5 m^3^. 

When it comes to preventing the transmission of SARS-CoV-2 and other airborne respiratory viruses, enhanced ventilation is a key factor to limit the spread of the virus because ventilation dilutes and removes infectious airborne droplet nuclei (aerosols). Ventilation control can be framed within a more general strategy for IAQ [16] that includes measures of parameters such as CO_2_ (a proxy for ventilation effectiveness), CO, NO_2_, VOCs, HCHO, and PM.

Currently, there are scarce works studying SARS-CoV-2 presence in indoor air. Likewise, the different sampling and analytical methodologies for detecting airborne viruses need to be assessed in an indoor air quality context. The main objective of this study is to fill the existing gap on this issue, and specifically: (i) to test different devices for sampling SARS-CoV-2 and other airborne viruses, (ii) to implement a new analytical process to detect respiratory viruses in the ambient air, and (iii) to Integrate airborne virus control in an IAQ context. 

## 2. Materials and Methods

### 2.1. Site Characterization

FISABIO-Mediterranean Ophthalmological Foundation (FOM) is an ophthalmological hospital where doctors perform general and specific eye tests to reach diagnoses and treat ocular pathologies. This ophthalmological hospital is located in one of the main avenues in the northwest of Valencia city (800,000 inhabitants). The center has more than 35 examination/rehabilitation rooms and three areas for operating rooms. 

The hospital’s ventilation system is based on ducts that allow indoor air to be expelled while outdoor air enters at a continuous flow. No room in the center has natural ventilation except the hall where the entrance doors are open throughout the working day. Additionally, HEPA filters are used in the ventilation ducts that reach the operating rooms.

**Figure 1:**
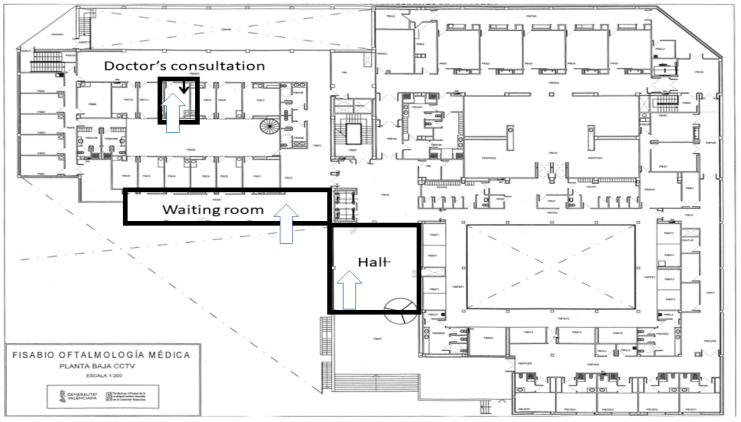
Map showing the sampling sites in the ophthalmological hospital: hall (2 doors, south orientation, 389.78 m^3^), waiting room (glazed wall and closed windows, south orientation, 281.46 m^3^), and doctor’s consultation (two doors, north orientation, 37.89 m^3^).

Three different areas around the hospital have been assessed during February 2021: (i) the hospital hall (space volume of 389.78 m^3^), (ii) the hospital waiting room (281.46 m^3^), and (iii) one doctor’s consultation room (37.89 m^3^). Each area was assessed once during a typical full day at the hospital. Figure 1 shows the location of these areas inside the building.

### 2.2. Air Sampling

Samples were collected in February 2021 (from 22nd to 24th) during Spain’s third COVID-19 pandemic wave [2]. Considering the literature [14,17], two different sampling procedures were chosen to assess the presence of airborne SARS-CoV-2 and other respiratory viruses in the indoor air: (i) a PTFE filter inserted in a 3-stage cassette and (ii) an MD-8 air scan sampler.

As regards the filter cassette, a PTFE filter inserted in a cassette (SKC, Pennsylvania, USA) was coupled to Lealand pump (SKC, Pennsylvania, USA) with a flow rate of 10 L/min, for 2.5 h, using a total air volume of 1500 L (1.5 m^3^) in each studied area (see Figure S1a). Total air volume collected was established according to Rahmani et al., 2020 [14]. One filter cassette was collected in each assessed room. 

Concerning the MD-8 air scan sampler (Sartorius Stedim Biotech, Aubargne, France), one gelatin filter with a nominal pore size of 3 µm, 80 mm in diameter, and soluble in water was employed (Sartorius Stedim Biotech, Aubargne, France). The two MD-8 air scans used for sampling viruses in the health center applied a flow rate of 50 L/min for 0.5 h (30 min) with a total air volume of 1500 L (1.5 m^3^) (see Figure S1b). The sampling conditions were set according to Ong et al., 2020 [3]. Two gelatin filters were collected in each assessed room. 

After the bioaerosol collection, filters were stored in zip bags, triple packaged (UN 3373, Biological Substance Category, type 3), and transported to FISABIO’s facilities, where the filter samples were immediately analyzed.

### 2.3. Surface Sampling

In order to investigate the presence of the SARS-CoV-2 on the hospital surfaces, certain surfaces were assessed in the different areas (one surface in each assessed room): (i) the protection screen used by the receptionist workers in the hall, (ii) the receptionist desk in the waiting room, and (iii) the doctor’s desk in the consultation room. In all cases, a commercial kit has been used (COV-Hygien Xpress on-site detection kit, VWR Avantor, Barcelona, Spain). 

The following describes the guidance on surface: a swab was employed, previously soaked with lysis buffer. Then, it was swabbed around the surface to be tested (15 cm^2^) and transferred to the test tube. After that, a strip was added in which there are two lines: one control line (it is necessary to see it to be sure that the test is valid) and one test line (if there is a visible color, the sample is positive; if not, the sample was negative). 

### 2.4. Indoor Air Quality (IAQ)

CO, CO_2_, HCHO, NO_2_, PM and VOC parameters have been assessed using sensors, Aeroqual Series 500 manufactured by Aeroqual HQ (Avondale, New Zealand). Table S1 shows the characteristics of the employed sensors. The temperature (°C) and relative humidity (RH) were recorded simultaneously for all instruments. Monitoring was conducted during the hospital’s busiest time of day (9:00 h to 13:00 h). The sensors were placed on tripods at a height of around 1.5 m to simulate a person’s breathing zone. These measuring instruments were in operation during virus sampling.

### 2.5. Sample Preparation for Analyzed Virus

A new analytical process has been employed to prepare air samples. The sample preparation for the cassette filters was as follows: 900 µL of RAV1 lysis buffer (Macherey-Nagel, Schkeuditz, Germany) was added to the cassette and gently shaken for two hours. Next, 750 µL was extracted and 5 µL of human DNA was added to the samples just before the extraction as an internal control for the quality of the extraction and amplification. Primers and probes for the RNAse-P gene were included in the real-time RT-PCR assays. 

For the gelatin filters (MD-8 air scan sampler), the employed sample preparation was the following: filters were cut with scissors and placed in 50 mL tubes. Then, 2 mL of lysis buffer RAV1 (Macherey-Nagel, Schkeuditz, Germany) was added to the tube that was heated for 5 min. The next step was to collect 200 µL from the tube and 550 µL of lysis buffer RAV1 and 30 µL of proteinase K (10 mg/mL), which were added to the sample. Then, the sample was heated for 10 min at 56 °C. Moreover, 5 µL of human DNA was added to samples just prior to extraction as internal control for the quality of the extraction and amplification.

In both cases, total nucleic acids were extracted with the Nucleospin-96 RNA kit (Macherey-Nagel, Schkeuditz, Germany) on an automated platform (Hamilton Starlet, Hamilton Company, Bonaduz, Switzerland). Each run included 1X phosphate buffered saline (PBS) as negative control and SARS-CoV-2 standard synthetic RNA transcripts containing 200 or 20 copies of targets E, N, ORF1ab, RdRP and S Genes of SARS-CoV-2 (EDX SARS-CoV-2 Standard, Exact Diagnostics LLC, Redmond, WA, USA).

### 2.6. Virus Analysis

#### 2.6.1. SARS-CoV-2 Detection by RT-PCR

A SARS-CoV-2 dual target (E and N genes) screening multiplex real-time PCR assay was performed in a Bio-Rad CFX (Bio-Rad, Redmond, WA, USA), using 8 µL of the eluted nucleic acid with the Vitro SARS-CoV-2 RT-PCR kit (Master Diagnostica, Granada, Spain). This assay also includes detection of the human RNAse-P gene in the multiplex, for sample collection, extraction, and amplification quality control.

To assess the efficiency and recoveries of the described methodology, samples tested in our laboratory facilities were spiked at different concentrations of SARS-CoV-2, commercially acquired at 200,000 copies/mL.

#### 2.6.2. Respiratory Virus Detection by RT-PCR

To detect the respiratory viruses, four different screening multiplex real-time PCR assays were performed in a Roche Lightcycler 480II apparatus, using 5 µL of the eluted nucleic acid for each assay with the qScript XLT One-Step RT-qPCR ToughMix (Quanta BioSciences, Gaithersburg, MD, USA). Multiplex 1 detected influenza virus type A and influenza virus type B, using probes designed for the Matrix protein in both cases [18,19]. Multiplex 2 detected human coronaviruses (HuCoV) 229E, NL63, OC43, and HKU1 by using probes from the 1b gene [20]; Human metapneuoviruses (HMPV) A and B by using probes from the N gene [21]; and Human bocavirus (hBoV), by using probes from the NP1 gene [22]. Multiplex 3 detected Parainfluenza viruses 1, 2, 3, and 4 using probes from the HN gene [23]; adenovirus (AdV), by using probes from the Hexon gene [23]; and Respiratory Syncitial viruses (RSV) A and B, by using probes from the NC gene [23]. Finally, multiplex 4 detected human rhinoviruses and enteroviruses (HRV/ENV), using probes from the 5′UTR region [24]. Laboratory procedures were strictly followed to prevent PCR contamination, and each run included positive purified viral nucleic acids as positive controls (AmpliRun DNA/RNA Amplification Controls Vircell, Granada, Spain) and negative controls (water without sample and/or nucleic acid).

### 2.7. Quality Control for Air Sampling and the Virus Detection

All equipment and materials used for this study were disposable, sterile, and RNAse/DNAse-free. Positive, negative, and internal controls were used as process quality control or quality assurance protocol in every analytical batch to guarantee that the respiratory viruses were analyzed properly. 

Moreover, we have collaborated in interlaboratory rounds organized by Ielab and LG for the SARS-COV-2 detection, obtaining satisfactory results. 

## 3. Results and Discussion

### 3.1. Previous Sampling Test

Before the sampling collection took place at the hospital, three different sampling methods (filter cassettes, MD-8 Air Scan, and Biosampler) for airborne viruses were tested in our laboratory facilities.

Most recent papers have preferred to use the Biosampler as a device for air sampling viruses like SARS or influenza [8,25]. This method bubbles the air through a liquid using an impinger [14]. Its pump (Biolite+, SKC, Johnstown, PA, USA) maintains a pressure of 0.5 atm, flowing the air through the nozzles and at a constant flow of 12.5 L/min. We tested four different collector liquids such as sterile distilled water, agarose culture medium, 1X PBS, and physiological saline solution (NaCl 0.9%). For each sampling collector liquid, the initial volume and the sampling time was optimized. Overall, the initial volume tested ranged from 5 to 10 mL and the sampling time was between 20 and 30 min. The problem we encountered with the sterile distilled water was that the final volume obtained for analysis was low due to evaporation. The PBS and agarose culture medium produced a large amount of foam, which meant sampling was unfeasible. The physiological saline solution was more appropriate, but we discarded the Biosampler device because the pump was very noisy and difficult to handle safely (a mobile glove box would be required to safely manage the samples during collection).

The other two samplers, the 3-stage cassette and the MD-8 air scan, used filters to collect the airborne viruses. Filters should have a pore size larger than viruses, and they offer high physical collection efficiency for virus-containing particles [17]. The filter’s material plays a key role in how effective it is for sampling the viral particle size [26,27].

In the present study, filter (PTFE) cassettes and gelatin filters (MD-8 Air Scan) were chosen due to the adequate conditions for carrying out an appropriate sampling and analysis of the viruses. Polytetrafluoroethylene (PTFE) membrane filters were employed in our study, as they have been in previous works [27,28] for sampling SARS and rhinovirus viruses in filter cassettes. PTFE has the advantage of not interfering with biochemical tests and the target viruses, and can be easily eluted from the membrane [29].

Regarding MD8 air scan sampling, this device has been used for sampling MERS-coronavirus (MERS-CoV) and SARS-CoV viruses in the indoor air of hospitals [30]. The MD8 air scan has also been previously employed for RNA virus collection, offering 100% collection efficiency [31], and has been used in Singapore hospitals to collect and detect SARS-CoV-2 viruses [3].

### 3.2. Airborne Viruses and Surface Results

Concerning the SARS-CoV-2 virus, it was not detected in the different areas of the eye hospital. Regarding to the obtained results, it is important to say that the RT-PCR is the most employed method in detecting SARS-CoV-2. However, an increase in digital PCR technique is expected in the next years, since it allows SARS-CoV-2 detection at lower concentration ranges [15].

In contrast to our findings, other studies also carried out in hospitals identified the presence of airborne SARS-CoV-2 virus, although most of these hospitals had COVID-19 patients. This was the case of a relevant study undertaken in different areas of two hospitals in China, one of which is exclusively dedicated to patients with severe COVID-19 and the other devoted to patients with mild COVID-19. Different concentrations of SARS-CoV-2 virus were detected in the ambient air of the hospitals, ranging from 1 to 42 copies/m^3^, both in areas with patients and in those exclusive to healthcare personnel, especially in the areas where staff change their clothing and personal protective equipment [9]. Furthermore, Kenarkoohi et al. (2020) [8] detected the presence of coronavirus in air samples in a hospital in Iran, remarking that the results reveal the possibility of airborne (aerosol) transmission of SARS-CoV-2 virus. On the other hand, and in accordance with our findings, Faridi et al. (2020) [32] did not detect the virus in air samples from another hospital in Iran. However, the authors did note the importance of further studies on aerosol transmission of SARS-CoV-2 virus.

The results of the other airborne respiratory viruses in the three assessed areas in the hospital are shown in Table 1. One respiratory virus normally associated with the common cold, human rhinovirus-enterovirus (HRV/ENV), was detected in the waiting room at low levels (Ct = 36.95), using the MD8 Air Scan. The measures taken to avoid the spread of SARS-CoV-2 virus, mainly the use of masks and proper ventilation, helps to avoid the presence of respiratory viruses in the indoor air.

Before the COVID pandemic, more airborne viruses were detected in different indoor environments. For instance, in 2017 and 2018, three different airborne viruses (influenza virus A, influenza virus D, and adenovirus) were detected in a hospital in Duke (NC, USA) [33]. Moreover, in a study carried out in a University Campus of Hong Kong, influenza virus A was detected in the air in a high frequency (16.4% of the samples). Influenza virus B and rhinovirus were also detected [34].

The survival and transmission of airborne viruses is highly related to the temperature and humidity. There is relationship between the survival of coronavirus and other airborne viruses, and the thermodynamic potential specific enthalpy of moist air, exhibiting a specific enthalpy around 55 kJ/kg-dry air, calculated combined temperature, and relative humidity in the assessed area [35]. Considering this, it is mandatory to control temperature and relative humidity in order to know more about airborne viruses.

Apart from airborne viruses, surface samples were also investigated for SARS-CoV-2. The obtained results in the assessed surfaces were negative in all cases, which is consistent with the absence of the virus in the indoor air. As in other works, SARS-CoV-2 virus was not detected, or only at a small percentage (<10%), on the assessed surfaces of the health centers, but in these cases, it was in areas with COVID-19 patients [36,37,38].

In contrast, other studies detected SARS-CoV-2 in more than 25% of the tested surfaces in hospitals [7,39].

### 3.3. Indoor Air Quality (IAQ)

To assess the IAQ, several chemical parameters were measured. Table 2 shows a summary of the obtained results.

Despite the heavy traffic around the hospital, CO levels in all areas were below the detection limit. Consequently, levels were lower than the exposure guideline value for 24 h in an indoor environment (8 mg/m^3^ = 7.03 ppm) established by WHO (2010) [40] and also lower than the new value established in September 2021 (4 mg/m^3^= 3.43 ppm) [41].

Other pollutants that come mainly from outside are nitrogen oxides, emitted by vehicular traffic and gas appliance for heating. NO_2_ concentrations detected in all assessed areas were very similar (around 0.05 ppm). These levels (see Figures S2–S4) were lower than the permissible exposure limit for NO_2_ of 0.5 ppm over an 8 h workday and a threshold exposure limit of 1 ppm [42].

As mentioned before, CO_2_, is a good proxy indicator of the ventilation rate. In our study, the highest levels of this pollutant were around 800 ppm and were observed in the consultation room (whose volume is small), as ventilation is limited and the doors tend to be closed. In the hall and the waiting room, values were below 700 ppm (see Figures S5–S7), which were lower than the 1000 ppm that the ASHRAE Standard 62-2010 [43] considers acceptable CO_2_ concentrations for appropriate air quality. However, this guideline value (1000 ppm) was set before the global pandemic situation. Given the evidence of airborne transmission of SARS-CoV-2, CO_2_, levels should not exceed 700 ppm because 1% of the air that a person breathes has already been inhaled by someone else in the space [44,45]. It is also important to mention that these concentrations were higher in the hall and the waiting room at specific times when there were more occupants in the hospital (around 11:00 a.m.). Consequently, CO_2_ concentrations in an indoor environment clearly depend on the number of occupants, the ventilation rate, the air exchange rate, and the room structure [46]. Moreover, high concentrations can be related to the presence of airborne viruses. Myatt et al. (2003) [27] suggested a positive relationship between the frequency of rhinovirus detected in the ambient air and the average concentrations of CO_2_. They concluded that higher CO_2_ concentrations were associated with an increased risk of exposure to potential infection. Consequently, it is important to maintain appropriate ventilation of the areas in order to avoid an increase in airborne virus levels.

**Figure 2:**
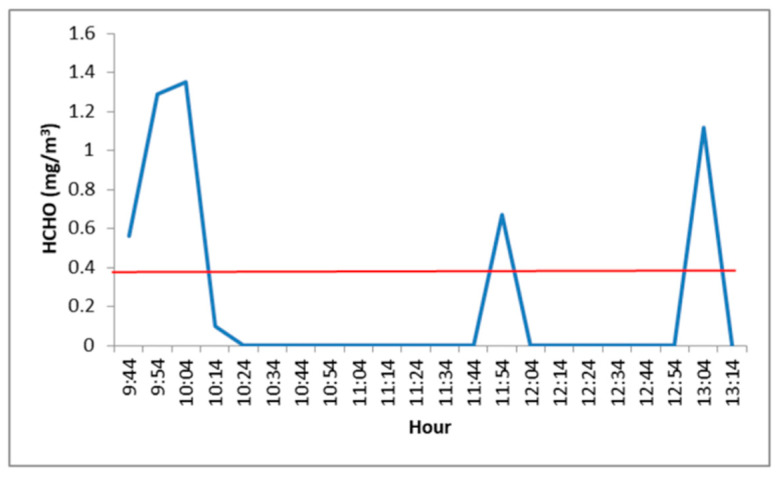
HCHO concentrations (mg/m^3^) in the doctor’s consultation (Red line: Guideline Value).

As to HCHO, it is used in the manufacture of sheet and insulation materials, primarily adhesives but also paints, cleaning agents, and cosmetics [46]. Formaldehyde levels in the hall and the waiting room were slightly higher than the guideline value (see Figures S8 and S9) in sporadic peaks (mainly at the beginning of the sampling period). Moreover, in the consultation room, these levels exceeded the guideline value at three moments (at the beginning, in the middle, and at the end of the sampling time frame) (see Figure 2). However, the obtained formaldehyde concentrations were, on average, lower than the guideline value over 8 h of exposure (0.3 ppm = 0.37 mg/m^3^) [42].

Indoor and outdoor sources can contribute to PM levels (PM_10_, PM_2.5_). Indoor PM is affected by ambient concentrations, air exchange rates, and penetration factors, as well as deposition and resuspension mechanisms. In all of the assessed areas, PM levels were lower than the established guidelines (see Figures S10–S15). Clean surfaces and floors help keep PM concentrations low, and ventilation by ducts also allows for a significant reduction of PM. According to WHO guidelines in 2010, the maximum concentrations in 24 h are 50 µg/m^3^ for PM_10_ and 25 µg/m^3^ for PM_2.5_ [40]. These values have been reduced in 2021 (45 µg/m^3^ for PM_10_ and 15 µg/m^3^ for PM_2.5_) [41].

**Figure 3:**
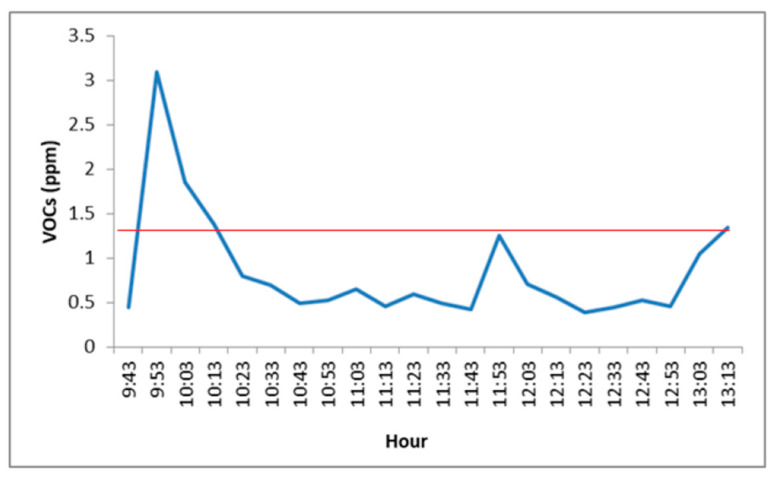
VOC concentrations (ppm) in the doctor’s consultation. (Red line: Guideline Value).

VOCs are widely present compounds in indoor environments as they are emitted from multiple internal and external sources. It is believed that increasing ventilation rates and using low-emission materials can improve their levels [47]. VOC levels in the doctor’s consultation room were found to be higher than the established guideline (1.32 ppm) at the beginning and end of the sampling period (see Figure 3). The other assessed areas (see Figures S16 and S17) presented lower levels than the guideline. VOC levels higher than 1.32 ppm (3 mg/m^3^) could cause concern regarding hygienic aspects [48].

According to INSHT guidelines for Spain [42], the temperature range in an indoor environment should vary between 17 °C and 27 °C and the relative humidity from 30% to 70%. Consequently, the obtained results (see Figures S18–S23) show that the assessed areas presented an appropriate temperature and relative humidity.

## 4. Study Limitations

The number of areas studied in the ophthalmological hospital could have been higher than the 3 studied areas in one week. Also, it would be better if more hospitals could be measured.

The RT-PCR could be insufficient to detect lower levels of SARS-CoV-2 in ambient air. The use of digital PCR technique could be able to detect SARS-CoV-2 at lower concentration ranges.

## 5. Conclusions

The methodology described in this work includes analysis of bioaerosols and IAQ assessment using sensors.

To detect airborne viruses, it is necessary to perform an appropriate sampling. Two sampling methods have been selected (filter cassette and MD-8 Air Scan) to collect SARS-CoV-2 in the ambient air. After that, a new analytical process using RT-PCR has been employed to detect 18 airborne respiratory viruses including SARS-CoV-2.

The developed methodology was applied in three different areas of an ophthalmological hospital in the Valencia Region of Spain. While SARS-CoV-2 virus was not detected in the indoor air of the assessed areas, nor on the surfaces of the hospital, one airborne respiratory virus (HRV/ENV) was detected in the waiting room. Thus, the findings show that this analytical process is suitable for detecting airborne viruses in the ambient air.

This assessment of bioaerosols was integrated with an assessment of the more conventional IAQ parameters. Whereas levels of CO, CO_2_, NO_2_, PM_10_, and PM_2.5_ presented acceptable values, VOC and formaldehyde levels were higher than the guideline values in the doctor’s consultation room at sporadic moments during the day. In summary, considering these results, the indoor air quality and bioaerosol levels of the ophthalmological hospital were found to be appropriate.

Finally, measurements of both chemical parameters and bioaerosols should be included in IAQ assessments, and the methodological issues described in this work can be used in programs focused on evaluating and promoting a healthy indoor environment.

## Figures and Tables

**Table 1:** Obtained results for respiratory virus in the indoor air.

Virus	Hall (Height = 0.8 m)	Waiting Room (Height = 0.7 m)	Doctor’s Consultation (Height = 1 m)
Influenza virus type A	Negative	Negative	Negative
Influenza virus type B	Negative	Negative	Negative
HuCoV 229E	Negative	Negative	Negative
HuCoV NL63	Negative	Negative	Negative
HuCoV OC43	Negative	Negative	Negative
HuCoV HKU1	Negative	Negative	Negative
HMPV-A	Negative	Negative	Negative
HMPV-B	Negative	Negative	Negative
hBoV	Negative	Negative	Negative
Parainfluenza virus 1	Negative	Negative	Negative
Parainfluenza virus 2	Negative	Negative	Negative
Parainfluenza virus 3	Negative	Negative	Negative
Parainfluenza virus 4	Negative	Negative	Negative
AdV	Negative	Negative	Negative
RSV-type A	Negative	Negative	Negative
RSV-type B	Negative	Negative	Negative
HRV/ENV	Negative	*Positive*	Negative

**Table 2:** Summary of the assessed IAQ parameters.

Parameter (Unities)	Range	Guideline Value
CO (ppm)	From < DL ^(1)^ to 0.68 ppm	7.03 ppm ^(5)^/3.43 ppm ^(6)^
CO_2_ (ppm)	From 526 ppm to 802 ppm	700 ppm ^(7)^
NO_2_ (ppm)	From 0.031 ppm to 0.059 ppm	0.5 ppm ^(8)^
VOCs (ppm)	From 0.17 ppm to 3.02 ppm	1.32 ppm ^(9)^
PM_10_ (mg/m^3^)	From < DL ^(2)^ mg/m^3^ to 0.005 mg/m^3^	0.05 mg/m^3 (5)^/0.045 mg/m^3 (6)^
PM_2.5_ (mg/m^3^)	From < DL ^(3)^ mg/m^3^ to 0.001 mg/m^3^	0.025 mg/m^3 (5)^/0.015 mg/m^3 (6)^
HCHO (mg/m^3^)	From < DL ^(4)^ mg/m^3^ to 1.35 mg/m^3^	0.37 mg/m^3 (8)^
Temperature (°C)	From 19.34 °C to 26.53 °C	From 17 °C to 27 °C ^(8)^
Humidity (%)	From 41.69 % to 49.89 %	From 30 % to 70 % ^(8)^

DL= Detection limit. ^(1)^ DL= 0.2 ppm. ^(2)^ DL= 0.001 mg/m^3. (3)^ DL= 0.0005 mg/m^3. (4)^ DL= 0.01 ppm. ^(5)^ WHO guidelines 2010. ^(6)^ WHO guidelines 2021. ^(7)^ LIFTEC and CSIC, 2020. ^(8)^ INSHT, 2021. ^(9)^ German Supreme Health Authorities, 2012.

## Supplementary Materials

The following are available online at https://www.mdpi.com/article/10.3390/toxics9110274/s1: Table S1, sensor specifications; Figure S1, sampling methodologies, (a) cassette and (b) AirScan with gelatin filter; Figure S2, NO_2_ concentrations (ppm) in the hall; Figure S3, NO_2_ concentrations (ppm) in the waiting room; Figure S4, NO_2_ concentrations (ppm) in the doctor’s consultation; Figure S5, CO_2_ concentrations (ppm) in the hall; Figure S6, CO_2_ concentrations (ppm) in the waiting room; Figure S7, CO_2_ concentrations (ppm) in the doctor’s consultation; Figure S8, HCHO concentrations (mg/m^3^) in the hall; Figure S9, HCHO concentrations (mg/m^3^) in the waiting room; Figure S10, PM_10_ concentrations (mg/m^3^) in the hall; Figure S11, PM_10_ concentrations (mg/m^3^) in the waiting room; Figure S12, PM_10_ concentrations (mg/m^3^) in the doctor’s consultation; Figure S13, PM_2.5_ concentrations (mg/m^3^) in the hall; Figure S14, PM_2.5_ concentrations (mg/m^3^) in the waiting room; Figure S15, PM_2.5_ concentrations (mg/m^3^) in the doctor’s consultation; Figure S16, VOC concentrations (ppm) in the hall; Figure S17, VOC concentrations (ppm) in the waiting room; Figure S18, temperature (°C) in the hall; Figure S19, temperature (°C) in the waiting room; Figure S20, temperature (°C) in the doctor’s consultation; Figure S21, relative humidity (%) in the hall; Figure S22, relative humidity (%) in the waiting room; Figure S23, relative humidity (%) in the doctor’s consultation.
