# Supplementary material for: Indoor Air Quality including Respiratory Viruses
Source: Toxics. 2021 Oct 21;9(11):274. doi: 10.3390/toxics9110274 (PMC8626032; doi:10.3390/toxics9110274)
Supplement: Supplementary file 1 [file toxics-09-00274-s001.zip › toxics-1412565-SI.pdf]

toxics

MDPI

Article

Supplementary Materials: Indoor Air Quality inclu1ding Respiratory Viruses

Antonio López, Esther Fuentes, Vicent Yusà, F. Xavier López-Labrador, Marisa Camaró, Cristina Peris-Martinez, Martín Llácer, Susana Ortolá and Clara Coscollà

Table of Contents

Type	Captions	Page
Table S-1	Sensor specifications	S3
Figure S-1	Sampling methodologies: a) Cassette, b) AirScan with gelatin filter	S4
Figure S-2	NO_2 concentrations (ppm) in the hall	S5
Figure S-3	NO_2 concentrations (ppm) in the waiting room	S6
Figure S-4	NO_2 concentrations (ppm) in the doctor's consultation	S7
Figure S-5	CO_2 concentrations (ppm) in the hall	S8
Figure S-6	CO_2 concentrations (ppm) in the waiting room	S9
Figure S-7	CO_2 concentrations (ppm) in the doctor's consultation	S10
Figure S-8	HCHO concentrations (mg/m^3) in the hall	S11
Figure S-9	HCHO concentrations (mg/m^3) in the waiting room	S12
Figure S-10	PM_{10} concentrations (mg/m^3) in the hall	S13
Figure S-11	PM_{10} concentrations (mg/m^3) in the waiting room	S14
Figure S-12	PM_{10} concentrations (mg/m^3) in the doctor's consultation	S15
Figure S-13	$PM_{2.5}$ concentrations (mg/m^3) in the hall	S16
Figure S-14	$PM_{2.5}$ concentrations (mg/m^3) in the waiting room	S17
Figure S-15	$PM_{2.5}$ concentrations (mg/m^3) in the doctor's consultation	S18
Figure S-16	VOCs concentrations (ppm) in the hall	S19
Figure S-17	VOCs concentrations (ppm) in the waiting room	S20
Figure S-18	Temperature (ºC) in the hall	S21
Figure S-19	Temperature (ºC) in the waiting room	S22
Figure S-20	Temperature (ºC) in the doctor's consultation	S23
Figure S-21	Relative humidity (%) in the hall	S24
Figure S-22	Relative humidity (%) in the waiting room	S25
Figure S-23	Relative humidity (%) in the doctor's consultation	S26

Table 1. Sensor specifications.

Gas	Sensor type	Work range (ppm)	Detection limit (ppm)	Resolution (ppm)	Operating conditions	
					Temp (ºC)	RH (%)
**CO**	**GSE[1]**	**0-100**	**0.2**	0.1	0-40	15-90
CO_2	NDIR[2]	0-5000	10	1	0-40	0-95
HCHO	GSE[1]	0-10	0.01	0.01	0-40	15-90
NO_2	GSE[1]	0-1	0.005	0.001	0-40	15-90
PM	LPC[4]	0.001-1.0 mg/m^3	0.001 mg/m^3	0.001 mg/m^3	0-40	0-90
VOCs	PID[3]	0-20	0.1	0.1	0-40	10-90

[1] GSE-Gas Sensitive Electrochemical, [2] NDIR-Non-dispersive infra-red, [3] pID- Photo Ionization Detector, [4] LPC- Laser particle counter

a)

b)

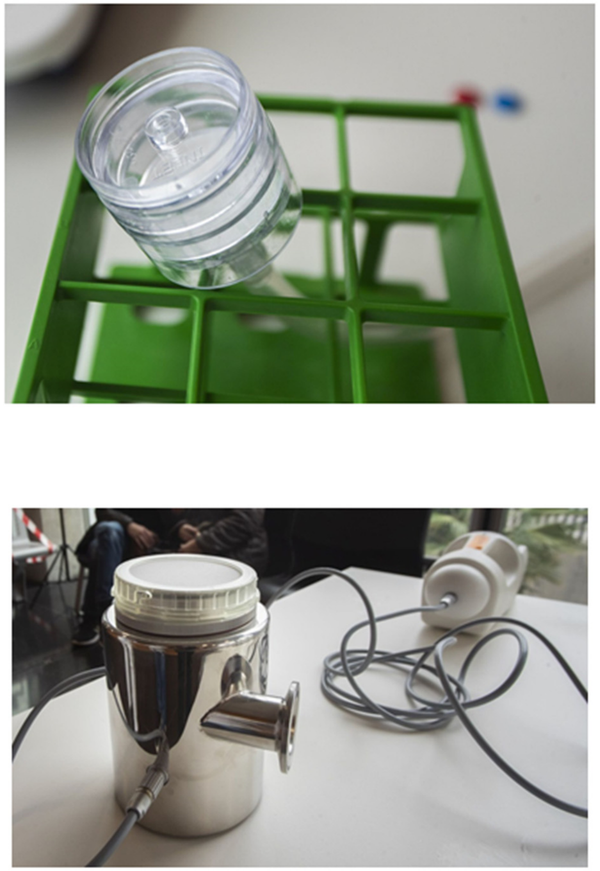

Figure 1. Sampling methodologies: a) Cassette b) AirScan with gelatin filter.

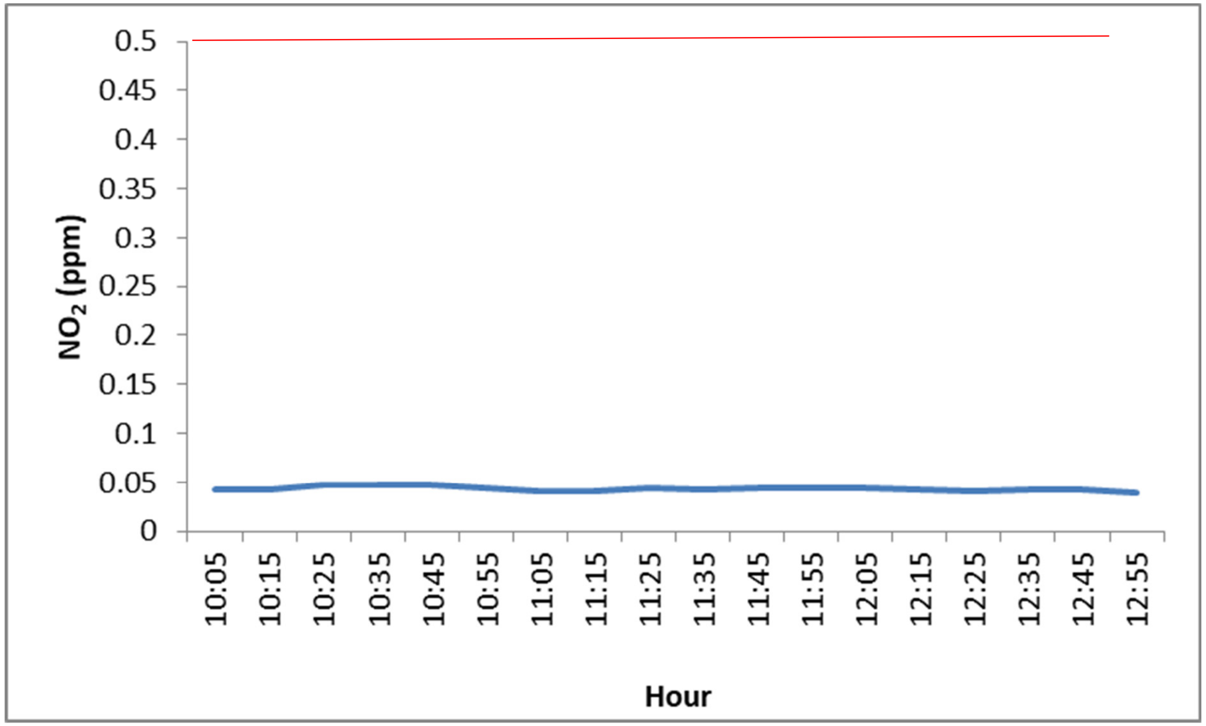

Figure 2. NO_2 concentrations (ppm) in the hall (Red line: Guideline value).

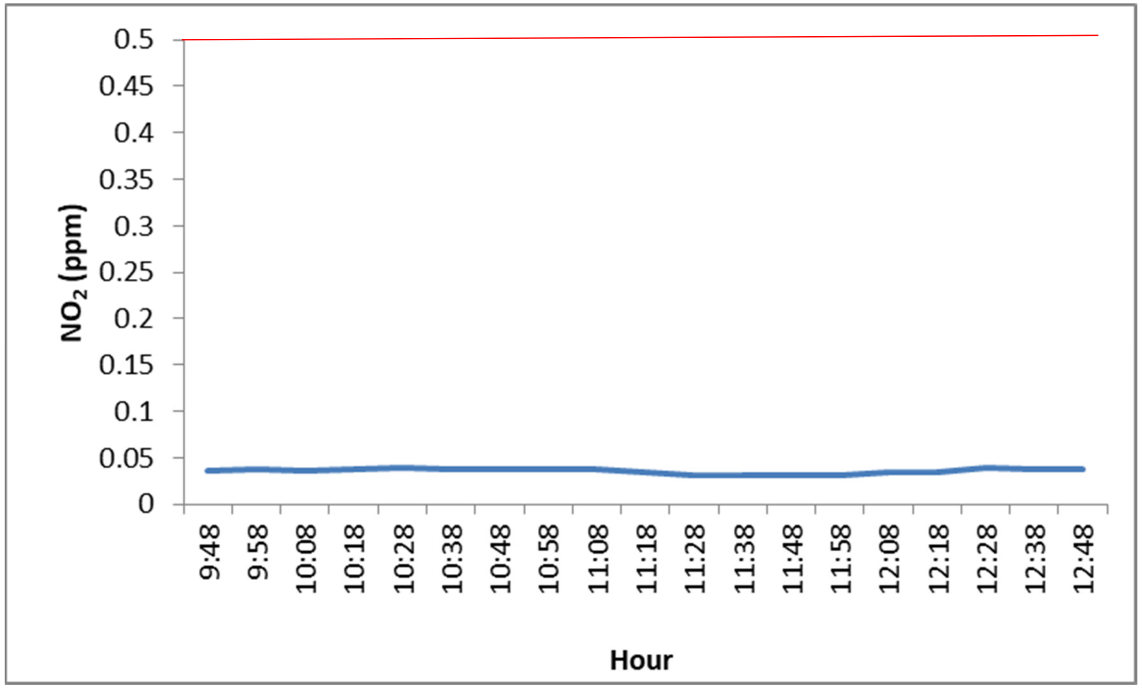

Figure 3. NO_2 concentrations (ppm) in the waiting room (Red line: Guideline value).

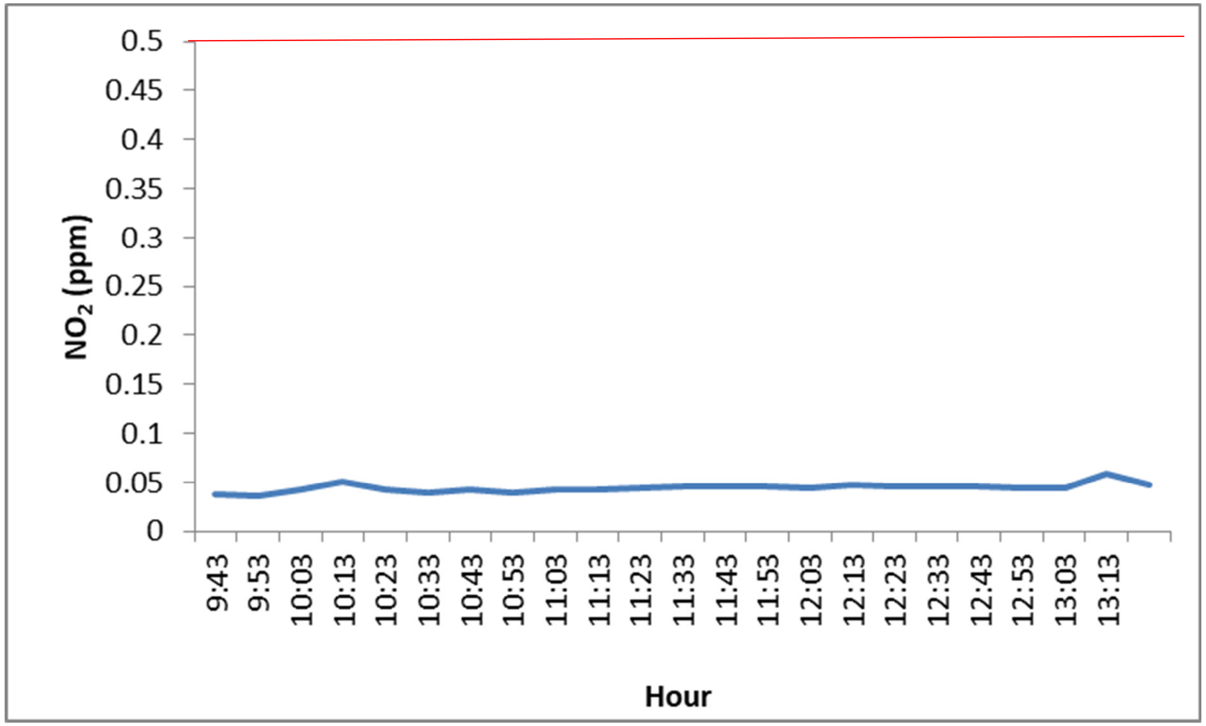

Figure 4. NO_2 concentrations (ppm) in the doctor's consultation (Red line: Guideline value).

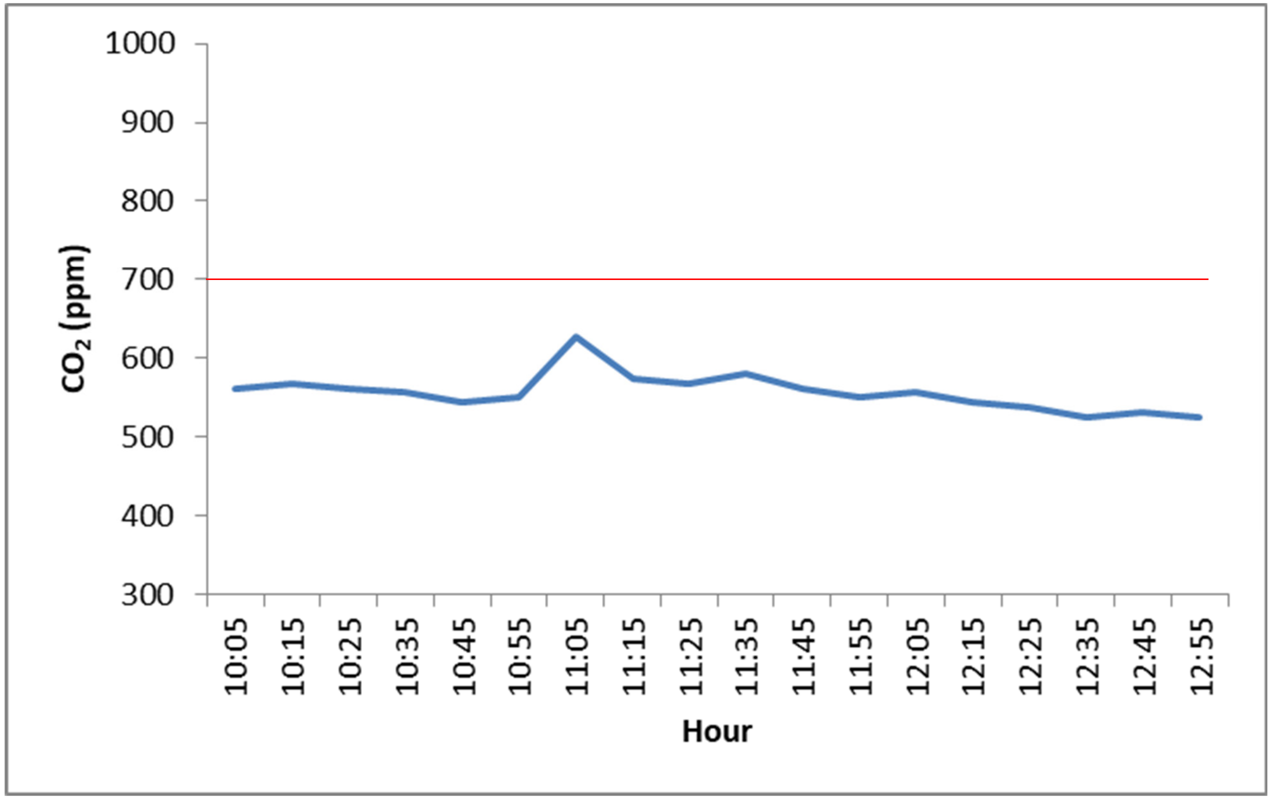

Figure 5. CO_2 concentrations (ppm) in the hall (Red line: Guideline value).

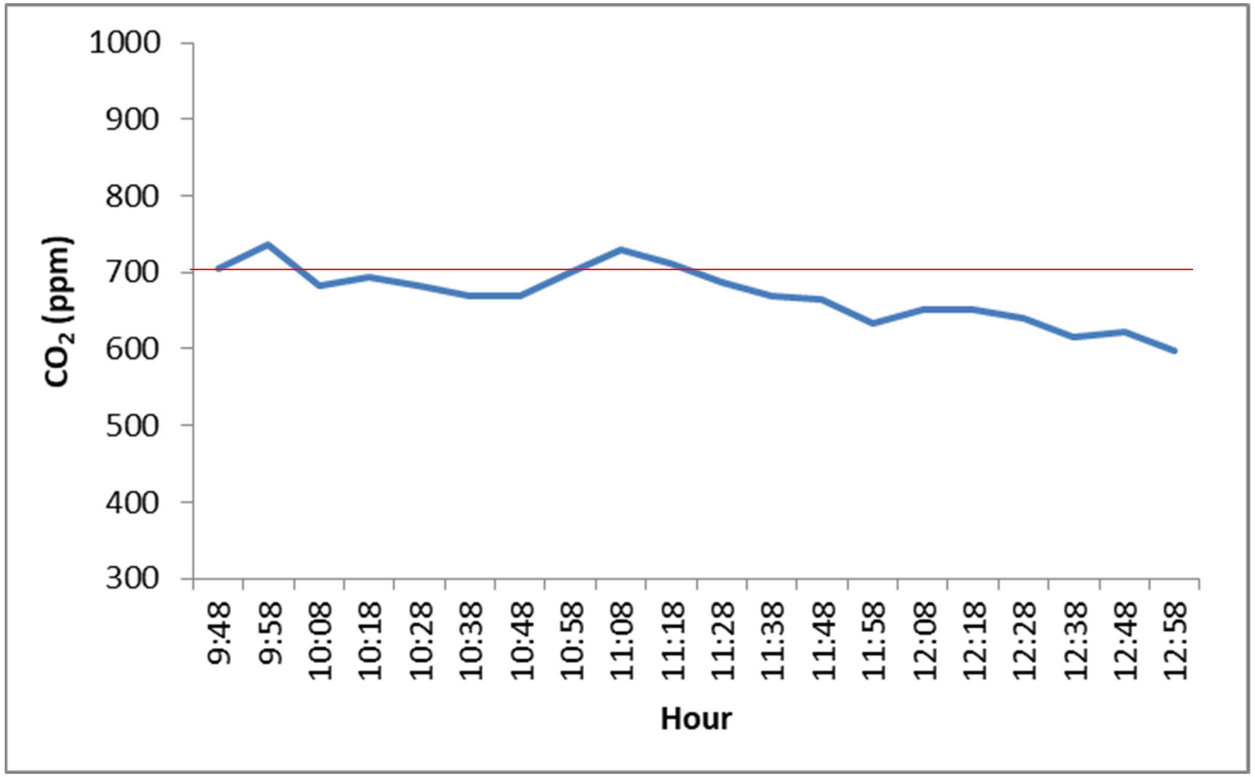

Figure 6. CO_2 concentrations (ppm) in the waiting room (Red line: Guideline value).

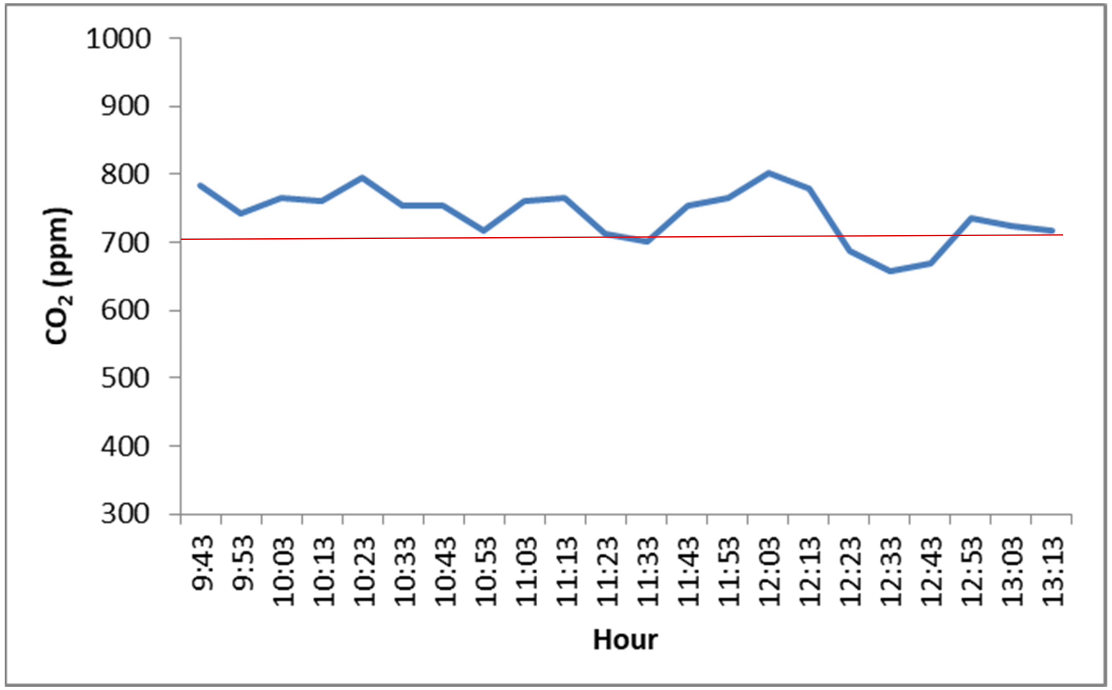

Figure 7. CO_2 concentrations (ppm) in the doctor's consultation (Red line: Guideline value).

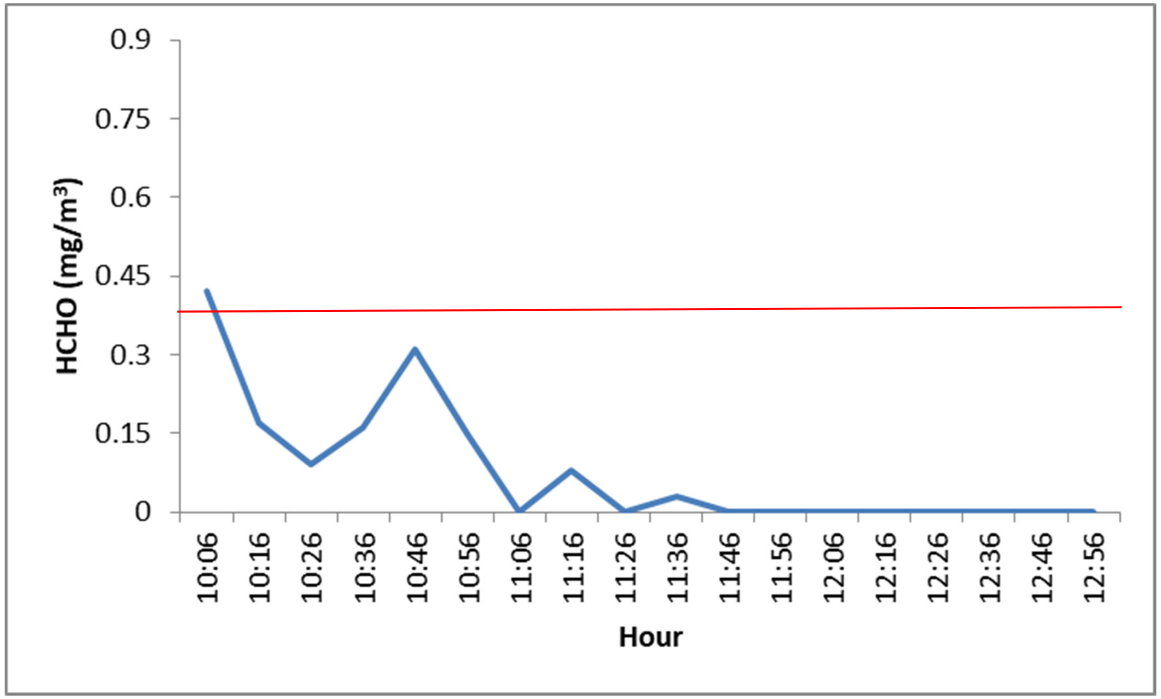

Figure S-8. HCHO concentrations (mg/m^3) in the hall (Red line: Guideline value).

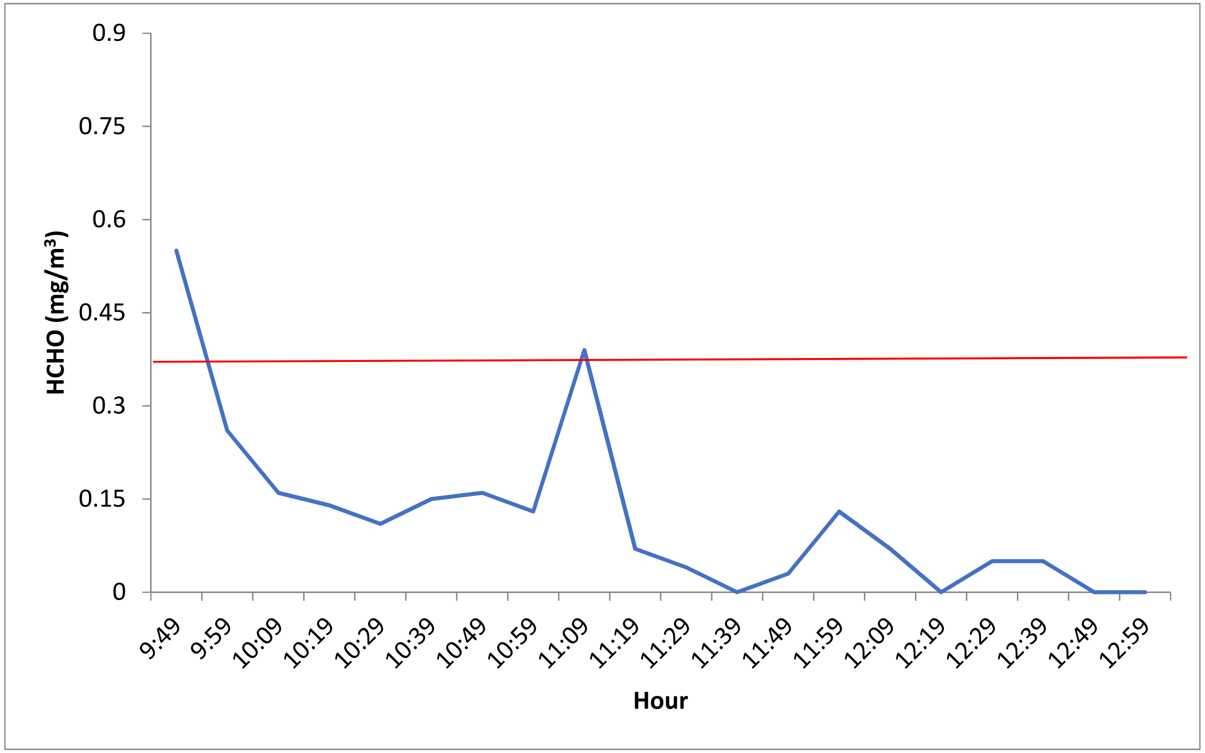

Figure 9. HCHO concentrations (mg/m[3]) in the waiting room (Red line: Guideline value).

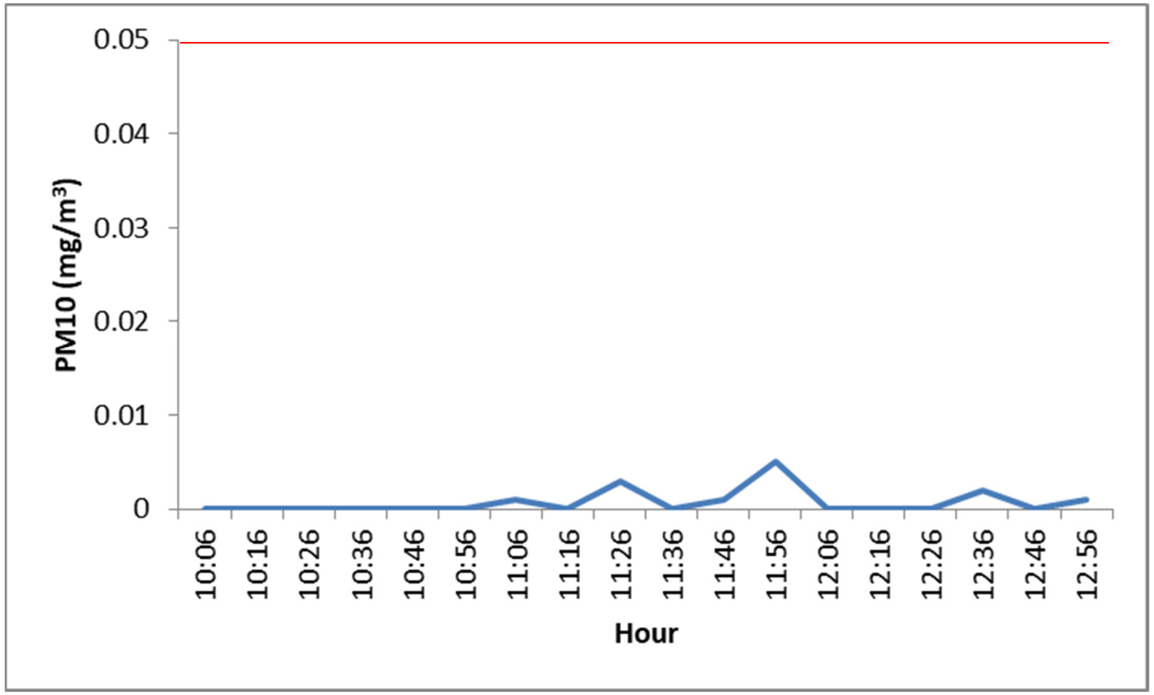

Figure 10. PM_{10} concentrations (mg/m^3) in the hall (Red line: Guideline value, WHO 2010).

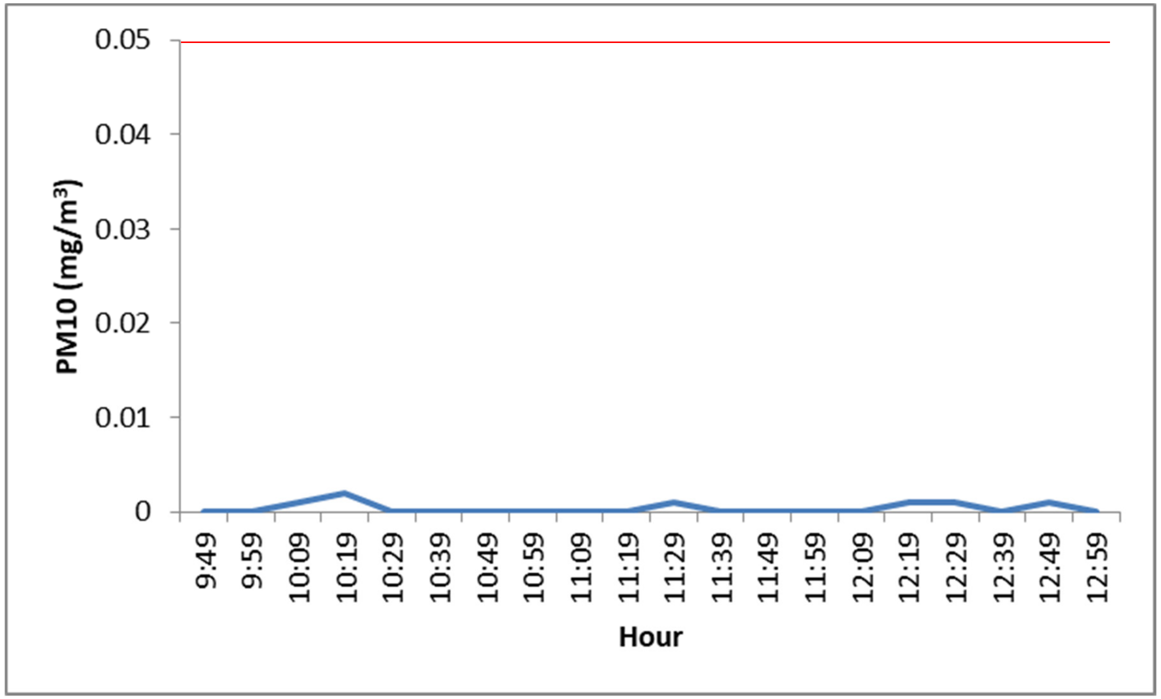

Figure 11. PM_{10} concentrations (mg/m^3) in the waiting room (Red line: Guideline value, WHO 2010).

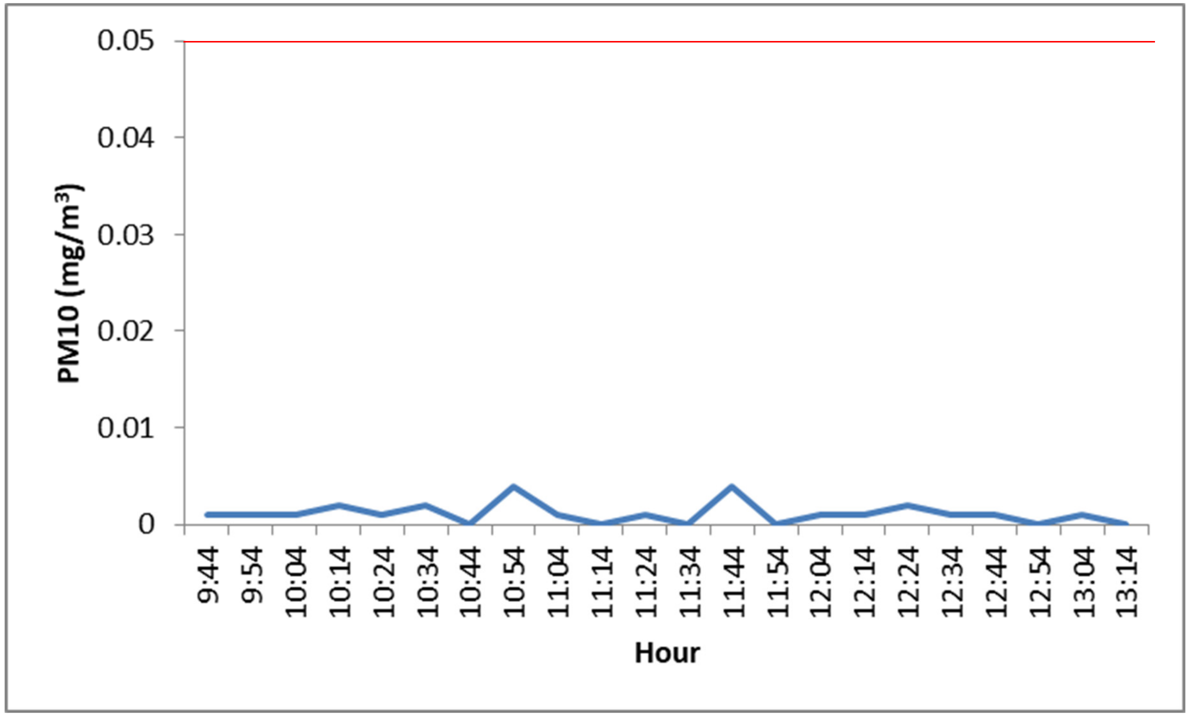

Figure 12. PM_{10} concentrations (mg/m^3) in the doctor's consultation (Red line: Guideline value, WHO 2010).

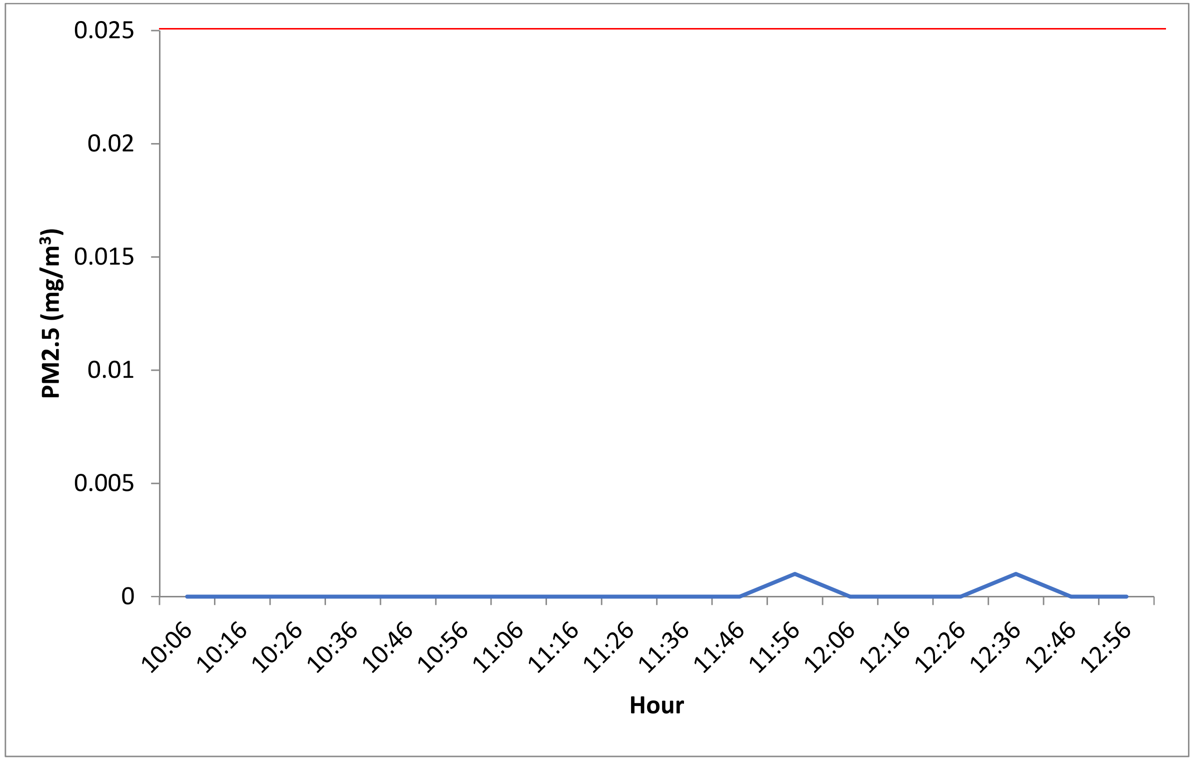

Figure 13. $PM_{2.5}$ concentrations (mg/m^3) in the hall (Red line: Guideline value, WHO 2010).

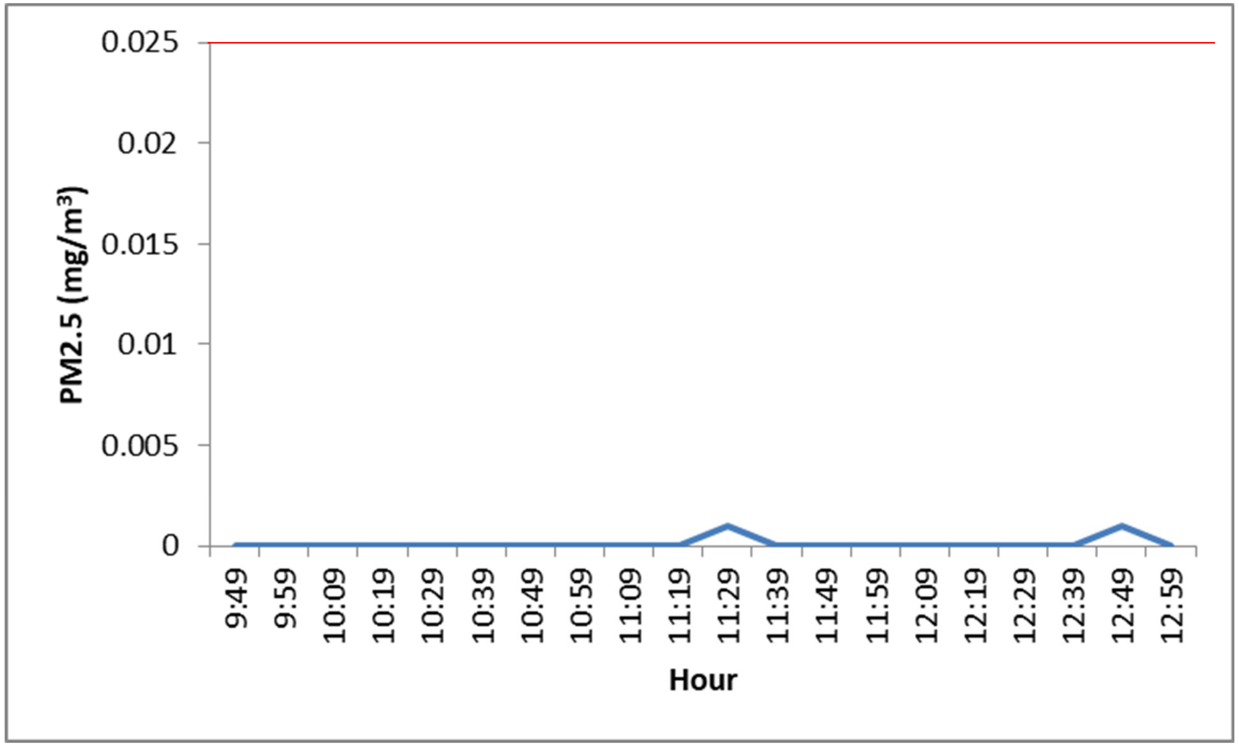

Figure 14. $PM_{2.5}$ concentrations (mg/m^3) in the waiting room (Red line: Guideline value, WHO 2010).

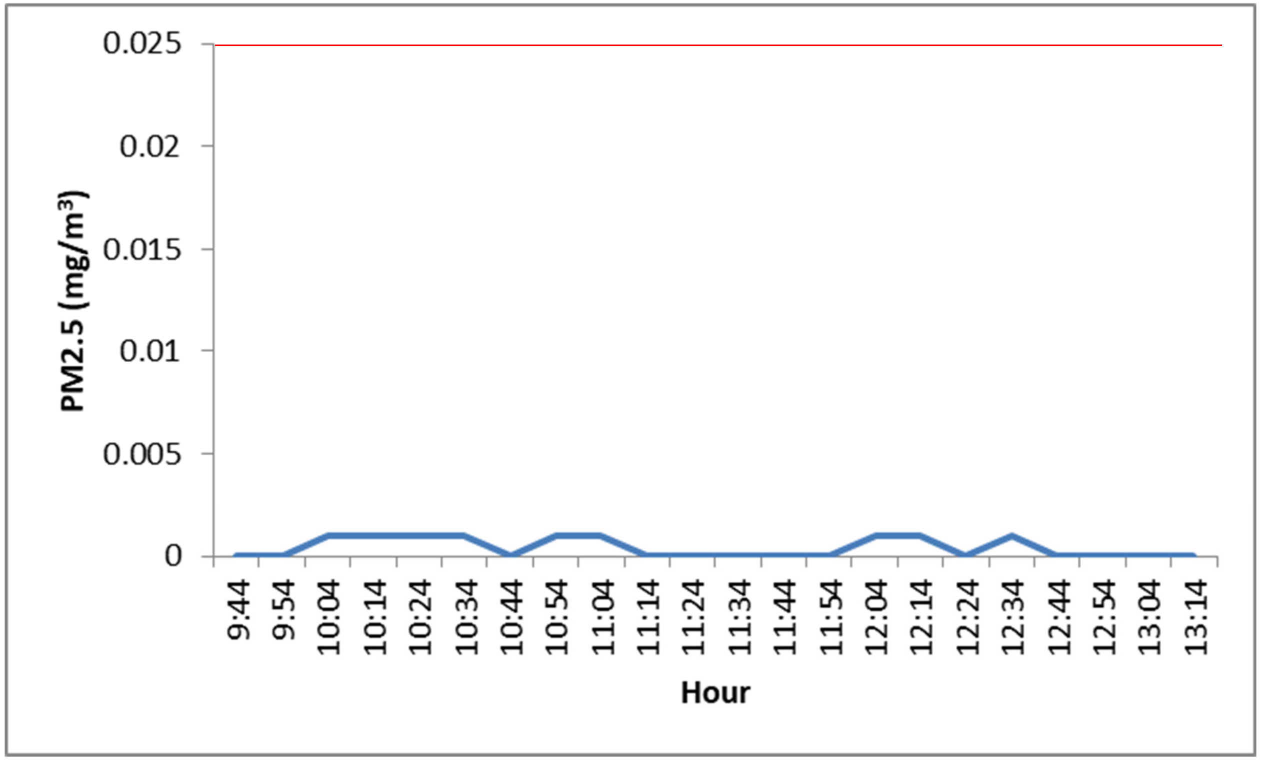

Figure 15. $PM_{2.5}$ concentrations (mg/m^3) in the doctor's consultation (Red line: Guideline value, WHO 2010).

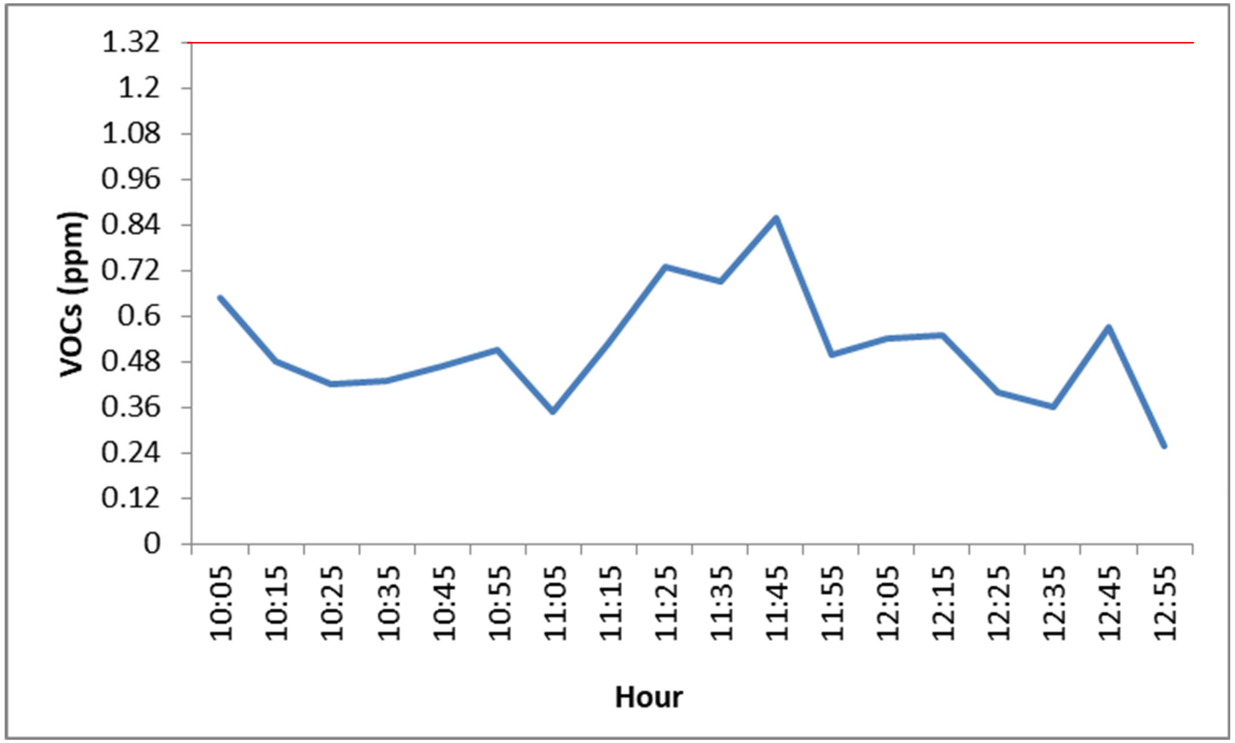

Figure 16. VOCs concentrations (ppm) in the hall (Red line: Guideline value).

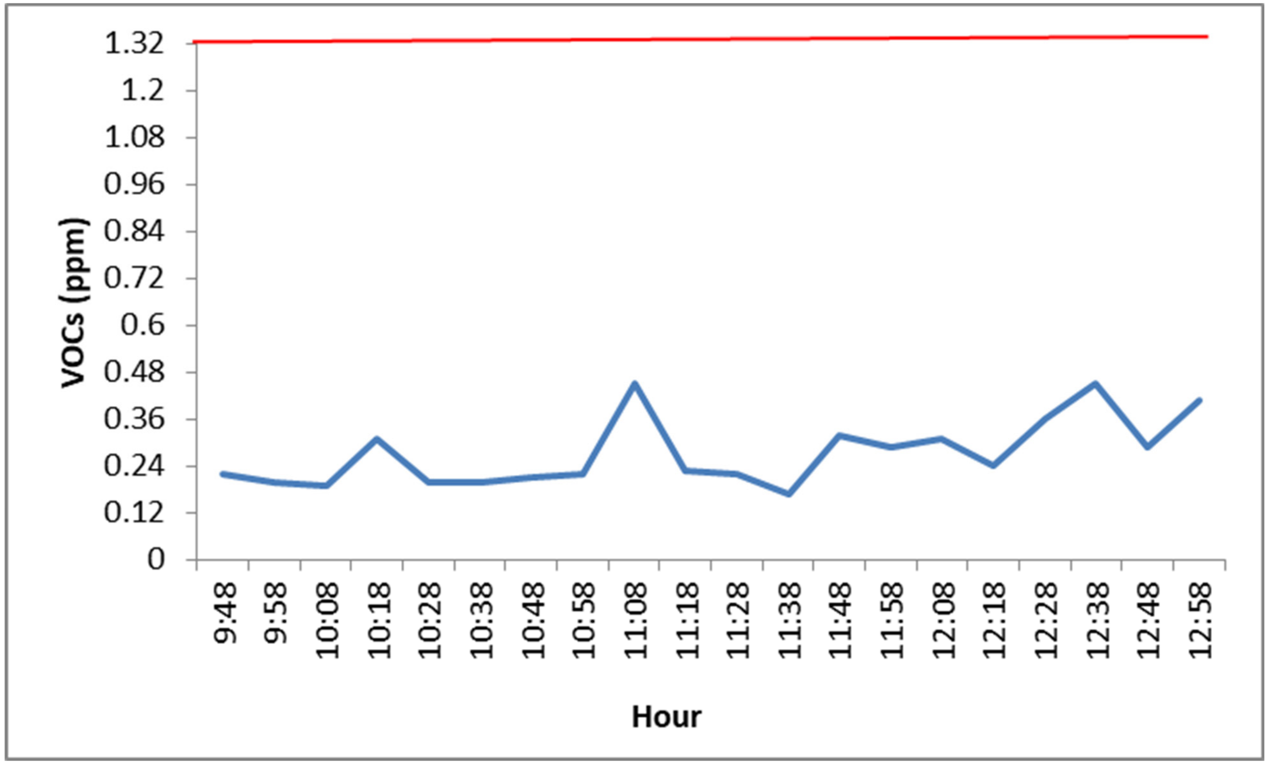

Figure 17. VOCs concentrations (ppm) in the waiting room (Red line: Guideline value).

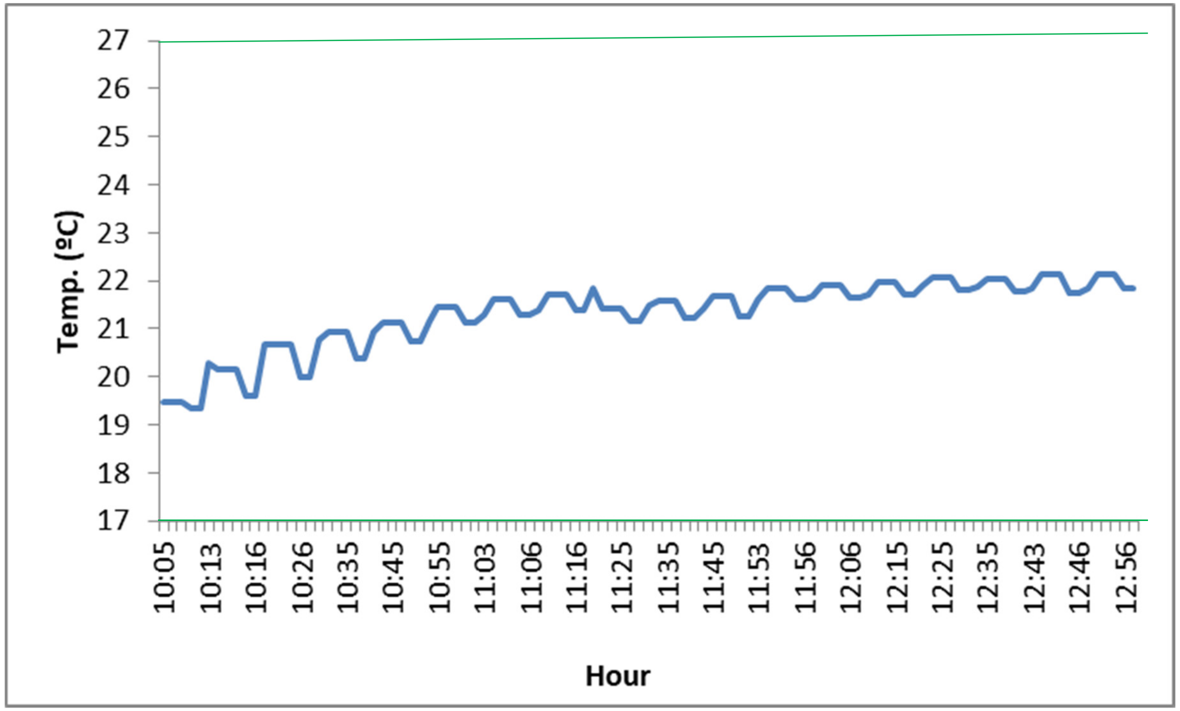

Figure 18. Temperature (ºC) in the hall (Green line: Appropriate range).

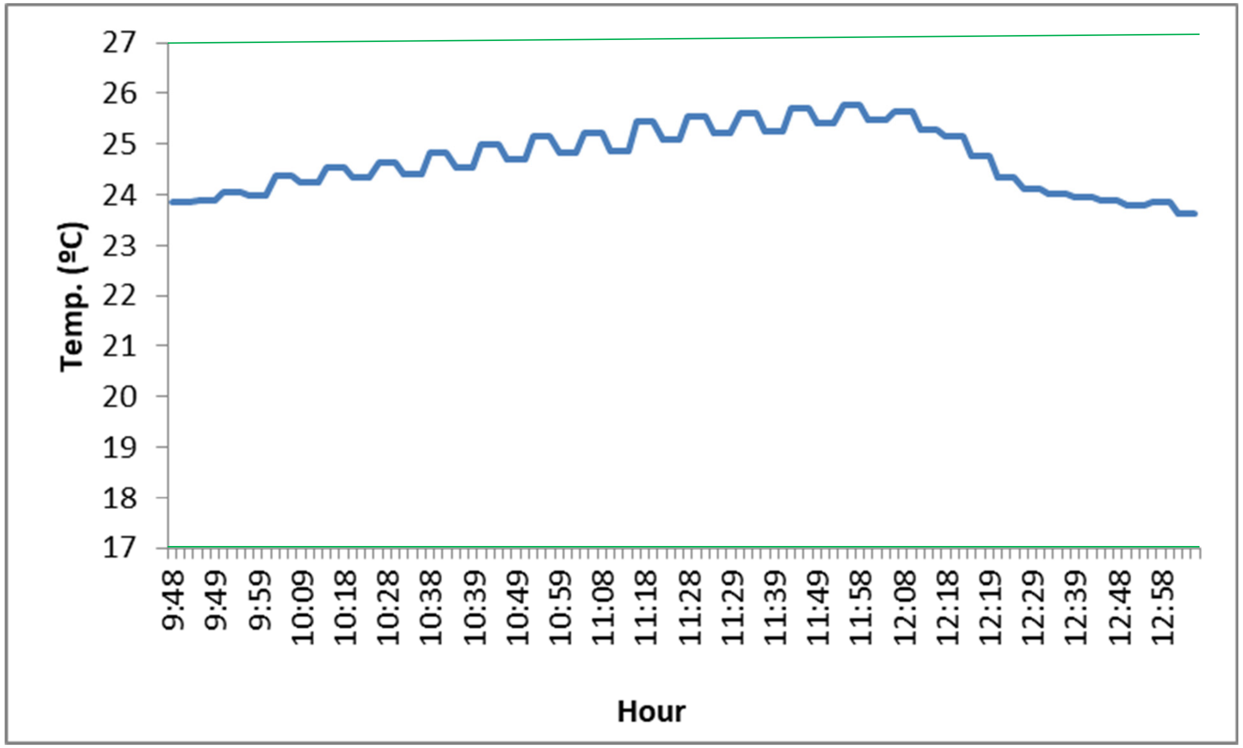

Figure 19. Temperature (ºC) in the waiting room (Green line: Appropriate range).

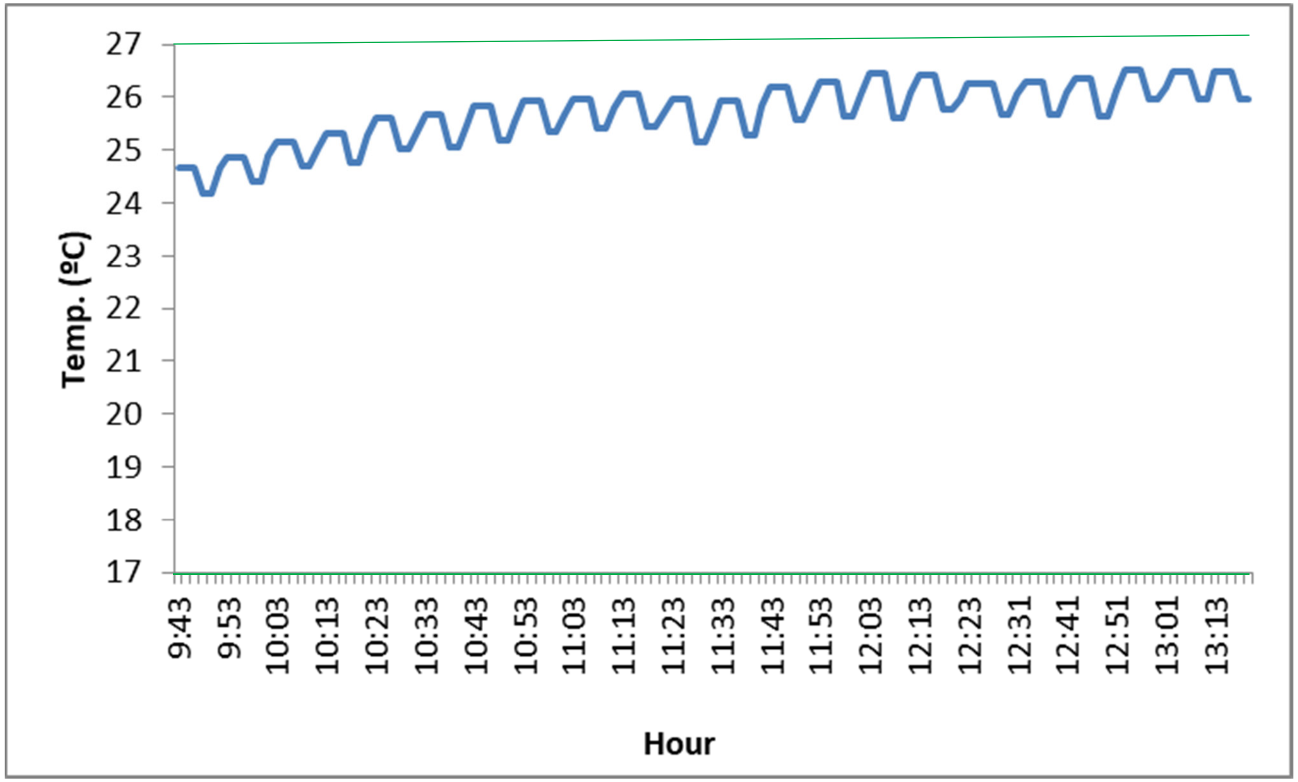

Figure 20. Temperature (ºC) in the doctor's consultation (Green line: Appropriate range).

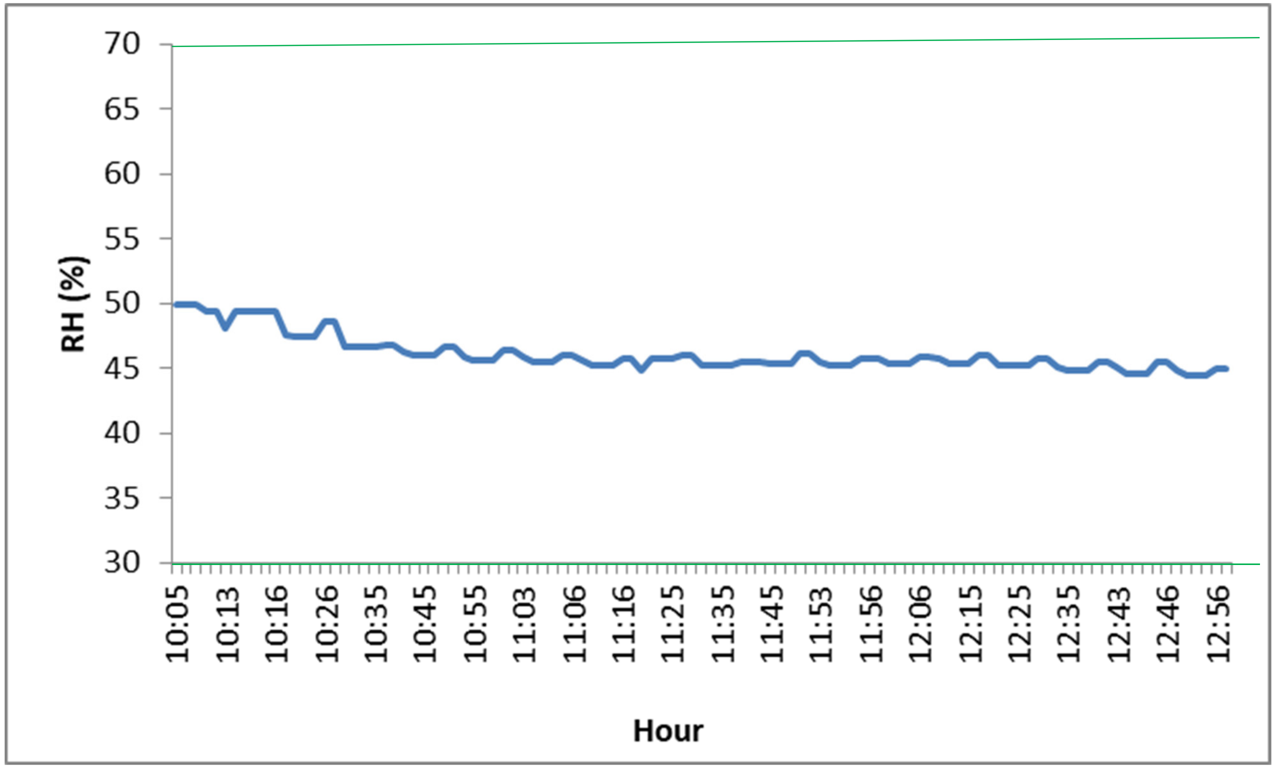

Figure 21. Relative humidity (%) in the hall (Green line: Appropriate range).

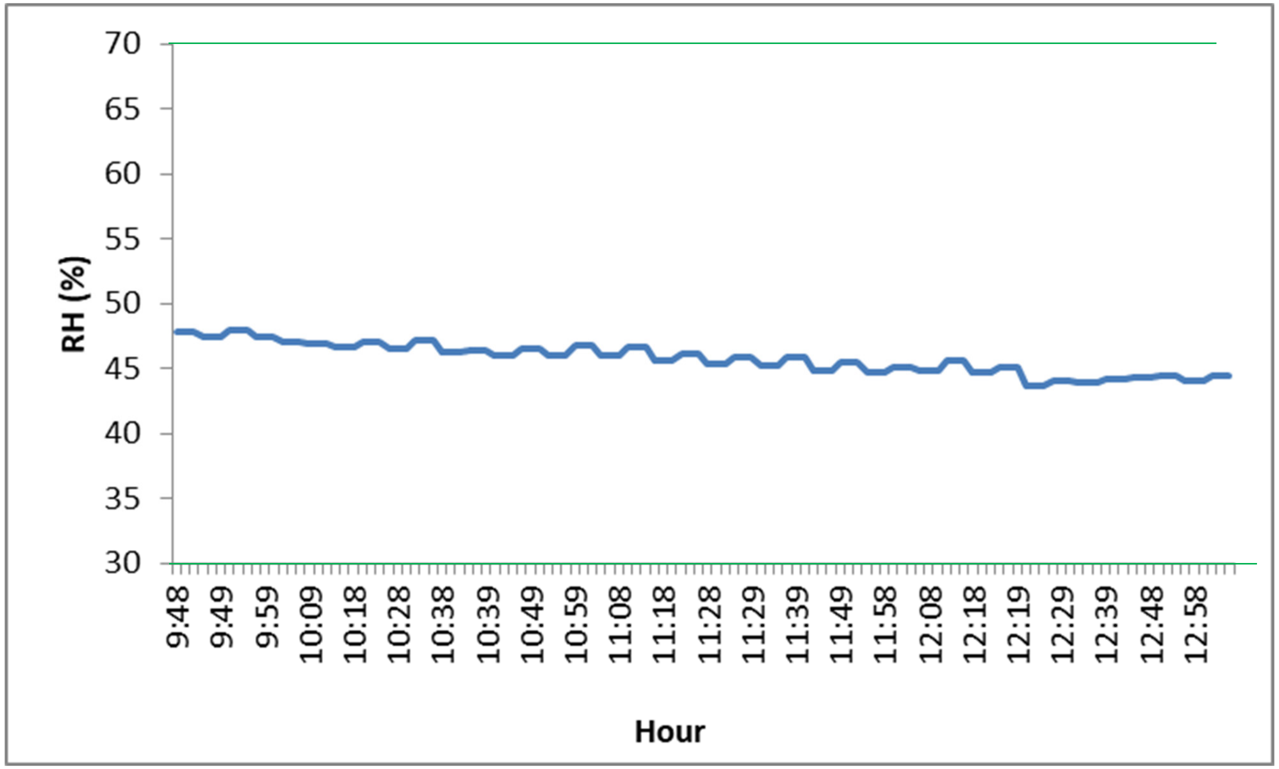

Figure 22. Relative humidity (%) in the waiting room (Green line: Appropriate range).

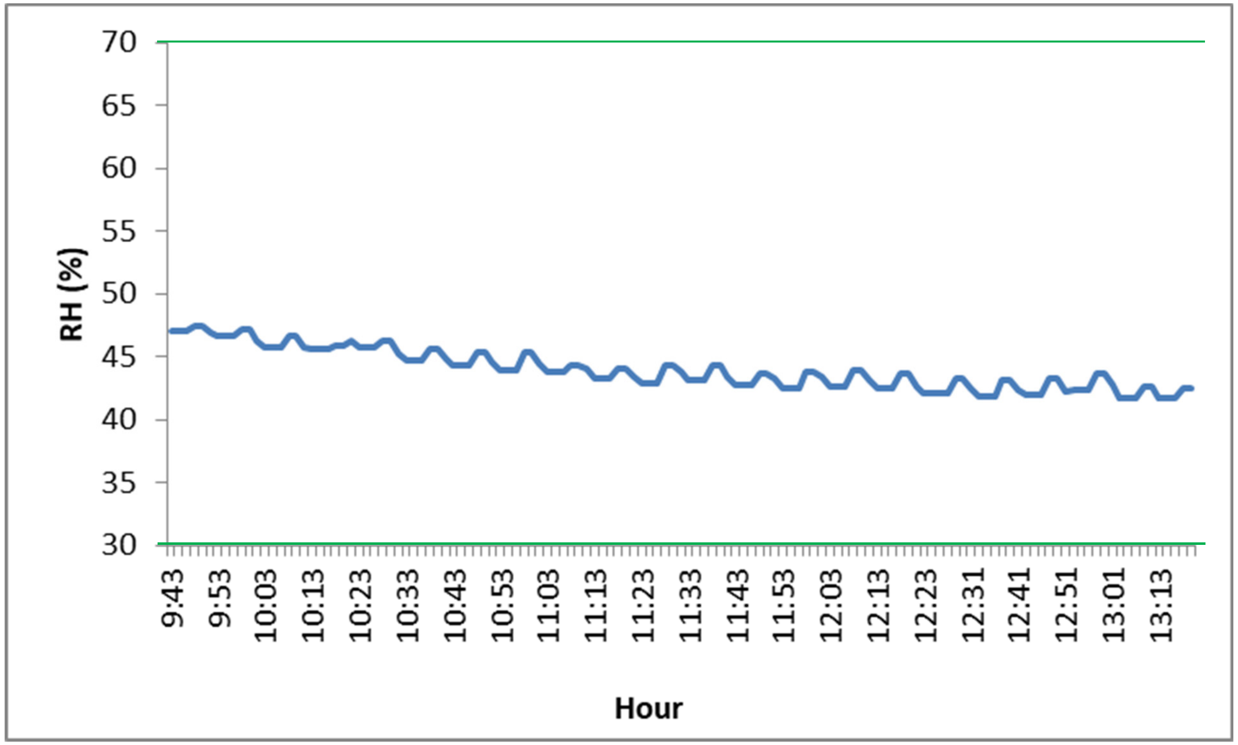

Figure 23. Relative humidity (%) in the doctor's consultation (Green line: Appropriate range).
